# Status Dystonicus, Oculogyric Crisis and Paroxysmal Dyskinesia in a 25 Year-Old Woman with a Novel *KCNMA1* Variant, K457E

**DOI:** 10.5334/tohm.549

**Published:** 2020-10-27

**Authors:** Cliona Buckley, Jennifer Williams, Tudor Munteanu, Mary King, Su Mi Park, Andrea L. Meredith, Timothy Lynch

**Affiliations:** 1 Department of Neurology, Dublin Neurological Institute, Mater Misericordiae University Hospital, Dublin, IE; 2 Temple Street Children’s University Hospital, University College Dublin, IE; 3 Department of Physiology, University of Maryland School of Medicine, Baltimore, Maryland, US; 4 Department of Health Affairs, University College Dublin, Dublin, IE

**Keywords:** Movement disorders, Genetics, Dystonia, Neurological Emergency, Seizure, Dyskinesia, PNKD3, BK channel, K_Ca_1.1, calcium-activated potassium channel

## Abstract

The diagnosis of a paroxysmal dyskinesia is difficult and status dystonicus is a rare life threatening movement disorder characterised by severe, frequent or continuous episodes of dystonic spasms. A 25 year old woman with chronic ataxia and paroxysmal dyskinesia presented with facial twitching, writhing of arms, oculogyric crisis and visual and auditory hallucinations. She developed respiratory failure and was ventilated. No cause was found so whole exome sequencing was performed and this revealed a novel, non-synonymous heterozygous variant in exon 11 of the *KCNMA1* gene, K457E (c 1369A>G) in the patient but not her parents. This variant has not been previously reported in gnomAD or ClinVar. The finding of a de novo variant in a potassium channel gene guided a trial of the potassium channel antagonist 3,4 diaminopyridine resulting in significant improvement, discharge from the intensive care unit and ultimately home.

## Introduction

Status dystonicus is a rare life threatening movement disorder characterised by severe, frequent or continuous episodes of dystonic spasms. It occurs in cerebral palsy, Wilson’s disease, mitochondrial disorders and new onset dystonic disorders and is usually triggered by infection, trauma, surgery or medication change including acute withdrawal from intrathecal baclofen or anticholinergics. Complications include bulbar paralysis, respiratory compromise, rhabdomyolysis, acute kidney injury, disseminated intravascular coagulation and death. The differential diagnosis of status dystonicus includes malignant hyperthermia, neuroleptic malignant syndrome and serotonin syndrome [1]. Paroxysmal dyskinesias are a group of uncommon disorders characterised by episodes of unpredictable movements which last for a brief duration and occur episodically [2]. We describe a patient with paroxysmal dyskinesia, ataxia, and subsequent status dystonicus with oculogyric crisis associated with a de novo genetic variant in the large conductance Ca^2+^ and voltage-activated K^+^ (BK, K_Ca_1.1) channel gene (*KCNMA1*) identified by next generation sequencing.

## Case Description

A 23-year-old woman, with intellectual disability, anxiety, paroxysmal dyskinesia and chronic ataxia, presented with vomiting, bilateral leg pain, difficulty mobilising and flexed posture of her neck. She recovered but represented at age 25 with a hyperkinetic movement disorder consisting of facial grimacing and perioral muscle twitching, writhing of upper limbs, and visual and auditory hallucinations. These symptoms were present for two days and were preceeded by a day of diarrhoea and vomiting two weeks previously. Medications on admission included alprazolam 500 μg tds, duloxetine 90 mg od and olanzapine 5 mg nocte for the treatment of a pre-existing anxiety disorder. In the emergency department she received haloperidol 2.5 mg intramuscularly for new onset visual and auditory hallucinations. She had no prior history of hallucinations. On day 2 of admission she developed respiratory failure and her Glasgow Coma Scale score fell to 3. The patient was intubated and ventilated. Olanzapine and haloperidol were stopped.

In the intensive care unit (ICU) she was hypertensive, tachycardic and pyrexic. There were hyperkinetic movements with posturing of the arms and legs and stimulus-sensitive fast twitching (?myokymia) of the upper lip, cheeks and eyebrows. Eye examination revealed recurrent upward deviation with intermittent downward eye movement to the mid position consistent with oculogyric crisis. There was bilateral inversion and plantar flexion of her feet with severe limb rigidity and mild axial rigidity (see Video 1). Hoffman sign was positive and there was symmetric hyperreflexia. She developed contractures of her hands and feet.

**Video 1:** **Examination of patient while intubated in the intensive care unit.** This video demonstrates stimulus sensitive involuntary movements of the lower limbs, bilateral inversion and plantar flexion of the feet with severe limb rigidity, mild axial rigidity and upward deviation of the eyes.

Full blood count, urea and electrolytes, liver and thyroid function tests were normal. C reactive protein was raised at 60 mg/L (<7 mg/L) and erythrocyte sedimentation rate was raised at 100 mm/hr (1–20 mm/hr). Creatine kinase levels were low at 18 I.U/l (33–208 I.U/l). Lactate levels were elevated at 3.6 mmol/l (0.5–2.2 mmol/l). Cerebrospinal fluid appearance was clear and colourless with 0 red cells and 0 white cells, glucose was 4.8 mmol/l and protein was 187 mg/L (150–450 mg/L). Microarray-based comparative genomic hybridisation (aCHG), APTX and FMR1 genetic testing were normal. Amino acid analysis was normal except for a slightly elevated asparagine at 55 umol/L (41–49 umol/L) and tyrosine at 119 umol/L (22–87 umol/L). Analysis for common mitochondrial DNA did not detect any abnormalities. Plasma acylcarnitine profile was normal. Organic acid analysis demonstrated a moderate increase in the excretion of 3-methylglutaconate and 3-methylglutarate. A muscle biopsy was normal. Urine catecholamines were all elevated including noradrenalin 1584 nmol/24H (0–900 nmol/24H), adrenalin 264 nmol/24H (0–230 nmol/24H), dopamine 3762 nmol/24H (0–3300 nmol/24H). Magnetic resonance imaging of the brain revealed previously identified cerebellar atrophy. CT thorax, abdomen and pelvis and whole body 18F-flurodeoxyglucose positron emission tomography were normal.

**Figure 1:**
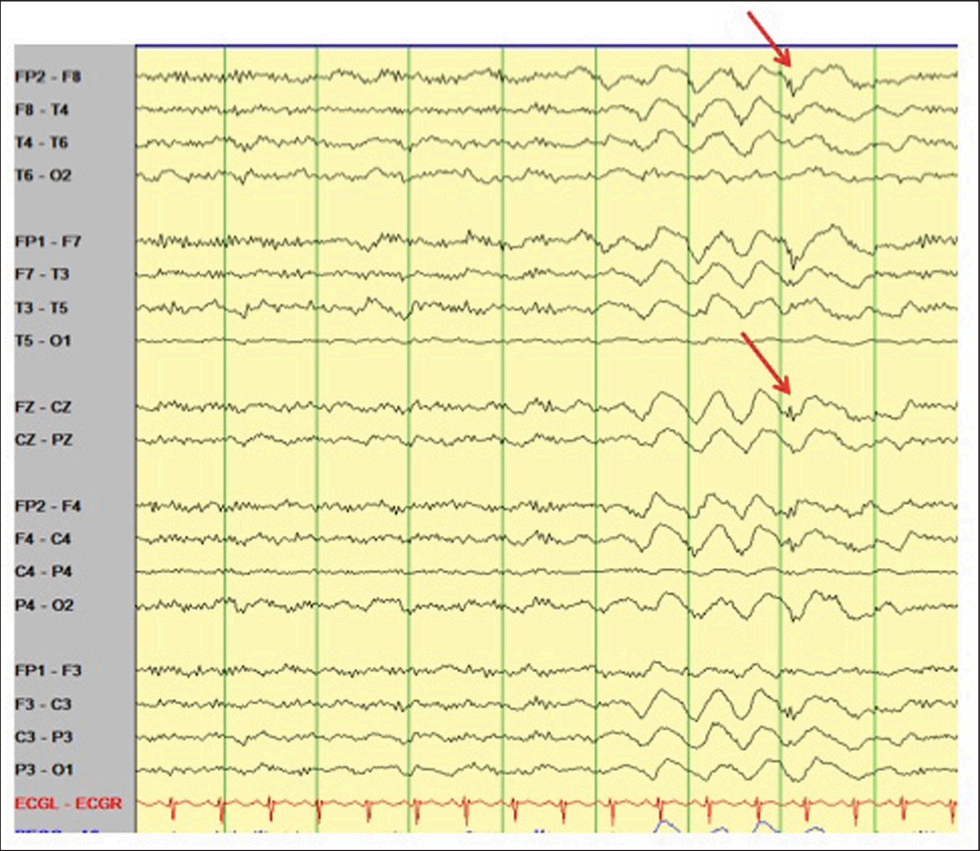
Demonstrating GRDA+S (red arrows).

An electroencephalogram (EEG) performed 12 months prior to presentation demonstrated normal background with no epileptiform abnormalities. Approximately 6 days into her ICU stay a routine portable study captured 1–1.5Hz 20–5-uV small amplitude poly-spike and slow wave activity without a clinical correlate. More prolonged video EEG monitoring while in ICU identified periods of 60–90 uV 1–2Hz generalized rhythmic delta activity (GRDA) at times with embedded spikes (GRDA+S) (Figure 1). Over a twelve hour period these periods of GRDA increased and subsequently the patient developed 2–2.5Hz rhythmic epileptiform activity over both frontal regions with abrupt onset, evolution and offset (Figure 2A and 2B). During the period of monitoring the patient has several electrographic seizures without clear clinical correlate. When the patient’s movements were most active there was no definitive EEG correlate. A follow-up EEG demonstrated lower amplitude posterior dominant rhythm but no epileptiform abnormalities.

**Figure 2A:**
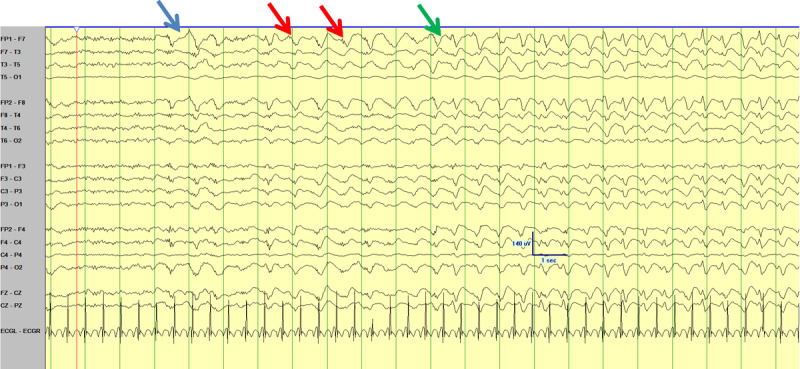
Demonstrating ictal onset (blue) of rhythmic bifrontal epileptiform sharp wave discharges in the average montage (red) with evolution (green) with a slight left sided lead in.

**Figure 2B:**
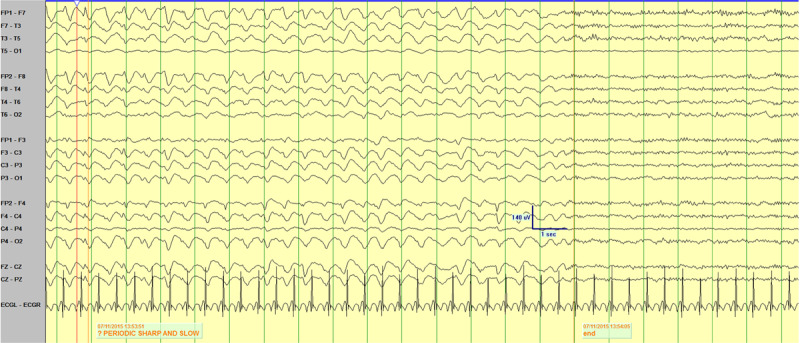
Demonstrating abrupt offset of rhythmic discharges.

Multiple medications were trialled including clonazepam, baclofen, diazepam, phenytoin, levetiracetam, lamotrigine, levodopa, oral baclofen and lacosamide. Intrathecal baclofen was added which resulted in improvement of rigidity but it had to be stopped as the patient developed bacterial meningitis. The patient intermittently reported visual hallucinations throughout the hospital admission. Further complications included ileus, ischaemic bowel and sepsis.

After many months in the ICU without a clear diagnosis, whole exome sequencing was performed. This revealed a novel, de novo, heterozygous genetic variant in exon 11 of *KCNMA1*, c 1369A>G in the patient. The potassium channel antagonist 3,4 diaminopyridine was added resulting in a marked clinical improvement allowing extubation, multiple medication decreases, transfer from ICU and ultimately discharged home sixteen months after admission. The patient is now dependent on others for all activities of daily living and has developed contractures of the upper and lower limbs. There have been no documented seizures since discharge.

## Discussion

The diagnosis and identification of the cause of paroxysmal dyskinesia can be difficult. Pathogenic alterations in ion channel activity can disrupt homeostatic and physiological functions leading to disorders such as hemiplegic migraine, epilepsy, cardiac arrhythmias or paroxysmal movement disorders, collectively known as “channelopathies” [4]. Paroxysmal nonkinesigenic dyskinesia, with or without generalized epilepsy (OMIM 609446; PNKD3) is associated with pathogenic variants in *KCNMA1*.

Moreover, status dystonicus is a life threatening condition associated with multiple causes. It requires urgent management and leads to significant mortality [1]. Identifying the cause for status dystonicus can guide therapy. Our finding of a single, de novo variant in a potassium channel gene, previously associated with a hyperkinetic movement disorder [3] in our patient with paroxysmal dyskinesia, oculogyric crisis and status dystonicus suggests this residue substitution should be considered with respect to causation of her symptoms.

*KCNMA1* encodes the alpha, pore-forming subunit of the calcium-sensitive potassium (BK) channel (OMIM: 600150) and is linked to the very rare autosomal dominant generalised epilepsy and paroxysmal non kinesigenic dyskinesia (OMIM: 609446 PNKD3) [3]. BK channels play an important role in neuronal excitability, passing outwards K^+^ current upon membrane depolarization and increased intracellular Ca2+ during the action potential, generally leading to hyperpolarization of the membrane and decreased excitability [3]. They are involved in the regulation of excitability in smooth muscle, neurons, neuroendocrine tissues and neurotransmitter release [3,5].

Sixteen *KCNMA1* variants in 37 symptomatic patients have been reported by Bailey et al. [3]. Our patient’s de novo variant (NM_001161352.2:c1369A>G; p.Lys457Glu) is novel and was not previously reported in the gnomAD (v2.1.1 and v3) or ClinVar databases. Since there are no data available from other patients harbouring de novo or inherited K457E variants, causality is uncertain. However, computational and functional data suggest this variant has the potential to be deleterious with respect to channel activity. Genome-wide pathogenicity predictor tools provide support for the deleterious potential of this missense residue change, assessed with Rare Exome Variant Ensemble Learner (REVEL score 0.645), Combined Annotation Dependent Depletion (CADD score 28.6), and Meta Logistic Regression (MetaLR score 0.3642) from the Ensembl Variant Effect Predictor (dbNSFP v4.1) and CADD v1.6. Seventy-five percent of pathogenic variants, but only 10% neutral variants, have a REVEL score >0.5 [6], and the variant falls within the top 1% of deleterious possible substitutions in the human genome by CADD score [7]. However, the less significant MetaLR score may be affected by the lack of allele frequency information [8].

Functional data provide further strong support for the deleterious nature of this variant. K457 is located in the αB helix of the first regulator of K^+^ conductance (RCK1) domain within the intracellular gating ring, suggesting the non-synonymous residue substitution could affect BK channel gating. The K457 residue interacts with Phosphatidylinositol 4,5-bisphosphate (PIP2), which facilitates BK channel activation. Neutralization of the K457 positive charge (K392N) decreased PIP2 sensitivity and also shifted the voltage-dependence of channel activation to more depolarized potentials, consistent with a reduction in channel function under these conditions [9]. K457 is also implicated in interactions with the β1 and β2 auxiliary subunits. Mutation of the lysine to glutamate (K392E, in tandem with an additional mutation, R393D) both increased the voltage required to activate the α-subunit alone and abolished the increased Ca^2+^ sensitivity normally produced by the β subunits [10,11]. Taken together, these electrophysiological studies support the hypothesis that the patient’s variant, K457E, produces loss-of-function effects on BK channel activity. Patients with other putative loss of function *KCNMA1* alleles also report epilepsy, developmental delay, various movement disorders (axial hypotonia, ataxia, paroxysmal dyskinesia and tremor), and cerebellar atrophy [3,5]. This patient’s presentation with longstanding ataxia, learning disability and acute status dystonicus, psychosis and oculogyric crisis is likely a representation of the phenotypic heterogeneity of *KCNMA1* variants.

At presentation, the patient experienced a new onset of hallucinations with no previous history of psychosis. It is possible that the psychosis was part of this patient’s acute illness associated with her KCNMA1 variant but a primary psychiatric cause cannot be excluded. Acute dystonic reactions, including oculogyric crisis, can occur with any dopamine receptor-blocking agents soon after the initiation of the medication [12]. Olanzapine and haloperidol are known to result in acute dystonic reactions including oculogyric crisis. Haloperidol administered for the treatment of her new onset hallucinations is associated with a high risk of acute dystonia [12]. However olanzapine was stopped following ICU admission and haloperidol was administered only once at the time of hospital admission. Considering the patient’s oculogyric crisis and status dystonicus persisted for months in ICU, it is unlikely that the administration of these agents were responsible for the hyperkinetic movement disorder or prolonged oculogyric crisis or status dystonicus.

## Conclusion

Paroxysmal dyskinesia and epilepsy associated with *KCNMA1* variants can respond to valproate, lamotrigine and partially to clonazepam [2]. Associated mutant BK channels are gain of function variants that increase voltage and calcium dependent activation [2]. Moreover, calcium channel antagonists may be a treatment option to counteract increased neuronal excitability. For example flunarizine reduces the severity and frequency of dystonic attacks in alternating hemiplegia of childhood [13]. However, no clinical evidence currently exists to support flunarizine’s use in *KCNMA1* variants. Our patient with a de novo probable loss-of-function *KCNMA1* variant associated with status dystonicus, oculogyric crisis, psychosis and longstanding intellectual disability, ataxia, and paroxysmal dyskinesia expands the phenotype associated with *KCNMA1* variants. Most importantly, judicial and cost-effective use of next generation sequencing can help make a diagnosis and guide effective treatment for critically ill patients with hyperkinetic movement disorders. Despite the prediction that K457E would decrease BK channel function, the potassium channel antagonist 3,4 diaminopyridine resulted in marked clinical improvement in our patient suggesting its inhibitory mechanism on K_V_ channels [14] does not contraindicate its use in patients harbouring *KCNMA1* variants of uncertain pathogenicity.
